# Polyandry in Noctuid Moths: Taxonomic, Bionomic, and Evolutionary Implications

**DOI:** 10.3390/insects16101063

**Published:** 2025-10-17

**Authors:** Zoltán Varga, Antal Nagy, Csenge Lelle Kovács, Szabolcs Szanyi

**Affiliations:** 1 Department of Evolutionary Zoology and Human Biology, Faculty of Science and Technology, University of Debrecen, H-4010 Debrecen, Hungary; varga.zoltan@science.unideb.hu; 2 HUN-REN-DE Conservation Biology Research Group, University of Debrecen, H-4010 Debrecen, Hungary; 3 Faculty of Agricultural and Food Science and Environmental Management, Institute of Plant Protection, University of Debrecen, H-4002 Debrecen, Hungary; nagyanti@agr.unideb.hu (A.N.); kovacs.csenge.lelle@agr.unideb.hu (C.L.K.)

**Keywords:** sex ratio, multiple paternity, spermatophore transfer, sperm competition, sexual selection, heterozygosity, pest species, genital asymmetry

## Abstract

Polyandry influences sexual behaviour and supports sexual selection in moths, incl. Noctuids. In polyandry, a female mates with multiple males within a few days. Copulation is a multi-phase process, as sperm are packed into spermatophores and transferred to the female genitalia. This process is costly, since spermatophore production and transfer is time-consuming. When this process is repeated, it results in sexual selection and enhances heterozygosity. Pest and migrating Noctuids are often polyandrous. Polyandry is common in the “pest clade” of Noctuidae (e.g., Euxoa, Dichagyris). We hypothesised that the asymmetric allocation of sensory and stimulating functions on the genitalia enhance sexual selection and promote speciation. In polyandrous species of the subtribe Poliina, we show that the evolution of the secondary asymmetry of male external genitalia is connected to an increase in species diversity. Some species inhabiting temperate forests were found to be moderately (Orthosia) or highly (Conistra) polyandrous, in connection with their different life cycles and despite similar reproductive timing. Based on fecundity data of some common species, we hypothesised that habitat generalists should be more polyandrous than habitat specialists. However, our data are insufficient to determine whether ecology and/or phylogeny influence the level of polyandry. Other factors, such as the connection between the sex ratio and the level of polyandry, should be surveyed in future studies, considering their relevance to plant protection.

## Simple Summary

Polyandry, i.e., multiple copulations of one female with several different males, promotes sexual selection in moths. Copulation is a multi-phase process, since sperm are packed into spermatophores and then transferred to the female genitalia. When copulation occurs repeatedly within a few days, it can result in sexual selection and enhance heterozygosity. Pest and migrating species are often observed to be polyandrous. Polyandry occurs regularly in several genera of Noctuid moths. Some economically important Noctuids of temperate forests are also moderately (Orthosia) or highly (Conistra) polyandrous, in connection with their different life cycles and despite similar reproductive timing. Based on fecundity data, we hypothesised that habitat generalists should be more polyandrous than habitat specialists. However, our data are insufficient to determine whether ecology and/or phylogeny influence the level of polyandry. Other factors of practical importance, such as the relationship between the sex ratio and the level of polyandry, should be examined in the future.

## 1. Introduction: Facts and Hypotheses Regarding Polyandry

Polyandry, i.e., multiple paternity, is widespread among animals and known in several groups of insects (e.g., grasshoppers, beetles, bees, butterflies, and moths) [1]. Previous studies have shown that polyandry promotes sexual selection [2,3,4,5,6], can influence sexual behaviour in experimental systems [7], and is a source of sexual conflicts [8]. Supposedly, a male-biased sex ratio is a pre-requisite of polyandry. In this case, a female often attracts multiple males, which can lead to multiple copulations within a few days and, consequently, sperm competition [9,10,11,12,13]. In lepidopterans, the act of mating is considered as male investment in the female by the transfer of a spermatophore, which is used for fertilisation and/or for nutrients, which have been found in the eggs and soma of females. Thus, spermatophores also function as nuptial gifts [14,15,16].

In Lepidoptera, copulation is costly, not only due to the male investment but also because it is a time-consuming, multi-phase process that often requires more than one hour [17,18,19] due to the intimate coupling of the external and internal genitalia of both sexes involving several specific co-evolved adaptations, often called “lock-and-key” mechanisms.

In polyandrous species, this multi-phase act is perpetuated several times within a few days. Since the average morphological distance between the genitalia of polyandrous species proves significantly larger than that of monandrous species [20], polyandry should support the co-evolving differentiation of genitalia during speciation, as hypothesised according to postmating sexual selection [20,21,22]. The costs of multiple investition should be compensated by some advantages in terms of fecundity and sexual selection [2,16,23]. It was shown that polyandry enhances heterozygosity and thus supports a sufficient level of genetic variation despite selection pressure [1]. Thus, polyandry can influence the population dynamics of numerous migrating, invasive, and economically significant species, as demonstrated in *Heliothis virescens, Helicoverpa armigera, Spodoptera spp., Busseola fusca, Pseudaletia unipuncta*, etc. [16,24,25,26,27,28]. Furthermore, a higher level of heterozygosity associated with polyandry can improve female fitness [5] and enhance the fertility, survivorship and competitivity of such species [29,30].

However, some recent studies have questioned both the direct and indirect genetic benefits of the multiple mating, i.e., polyandry [31], as hypotheses and inferences regarding polyandry and its consequences have mostly been based on laboratory experiments, e.g., [18,32,33,34,35]. For practical insights, we should uncover how polyandry occurs and functions in natural populations. In other words, we should determine how the observed male-biased sex ratio, as a possible prerequisite of polyandry, is achieved in natural populations of different polyandrous species. In the following sections, we state some hypotheses on the connection between life history and polyandry in moths and describe our attempts to test these hypotheses on some genera of Noctuidae. We also describe suitable and cost-efficient tools to detect and analyse polyandry-related processes in natural populations of moths, particularly pest species.

## 2. Materials and Methods

Moths were collected using light and pheromone traps, and collection specimens were dissected following standard methods of genitalia preparation [36] with some modifications [37]. Potassium hydroxide (15% KOH solution) was used to macerate the whole abdomen. In female specimens, the fat bodies were removed by a short treatment with cc. acetic acid. The bursa copulatrix, ductus, and ovipositor were carefully cleaned, sparing the spermatophores in the bursa. The bursa was then inflated by injecting 96% ethanol and moderately stained with chlorazol black. In males, the cleaned genital capsule and the everted and inflated vesica were dehydrated in 96% ethanol, and the weakly sclerotised structures were stained with chlorazol black.

For museum specimens and other voucher individuals, we prepared permanent slides of genitalia mounted to Euparal. The mounted slides were digitalised with a GT Vision PrimeScan Microscope Slide Scanner (*Giffords Ln, Newmarket CB8 8PQ, UK*). Genitalia terminology followed the standards of the Noctuidae volumes of MONA (Moths of North America).

## 3. Hypotheses and Results

### 3.1. Trade-Offs in Copulation: Structures, Functions, and New Working Hypotheses

In Lepidoptera, copulation is associated with the formation and transfer of spermatophores. This is a time-consuming process and consists of several pre-mating steps: (i) the perception of female pheromones (long-distance effect); (ii) the production of male pheromones (side-by-side effect); and (iii) stimulation by, and mechanical association, with the external genitalia [18,38]. Spermatophore production and transfer are multi-phase acts requiring long, intimate contact between both the external and internal genitalia of males and females, as has been demonstrated in different pest species (*Heliothis zea* [39], *Peridroma margaritosa, Pseudaletia unipuncta* [17,34]; *Spodoptera litura* [18]). These steps are performed by a complex system involving co-evolved components of the male/female genitalia, referred to as “lock-and-key” mechanisms [40,41].

In describing the steps of copulation, we generally follow the nomenclature suggested by Mikkola [40], distinguishing the external genitalia and coupling from the internal genitalia and coupling and defining the lock-and-key mechanisms as morphological correspondences in the genitalia of the two sexes; however we also include the sensory structures that promote correct nesting of the morphological structures.

Therefore, we must determine how system operates during the premating and mating steps. We answered this question in different ways, in which we consider two alternative explanations in connection with the lock-and-key mechanism.

**Figure 1:**
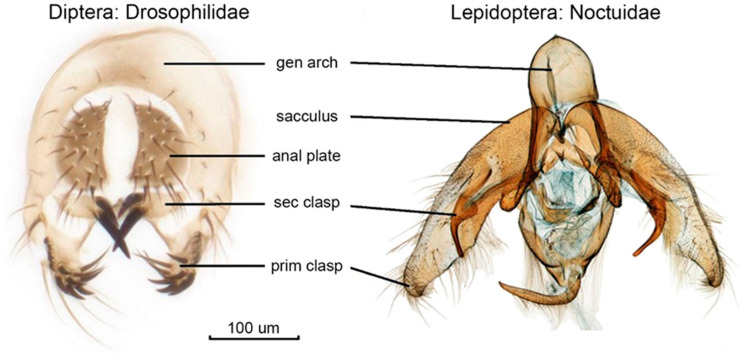
Male genitalia of *Drosophila* sp. (simplified) and of *Rhyacia psammia* (Noctuidae), with homologies of clasping structures. Explanation of the abbreviations: gen. arch, saccus; sec. clasp, harpe (clasper); prim. clasp, valve with corona bristles (*Rh. psammia*: slide VZ7367, Iran, Zaghros Mts, Sendan).

(i) According to the “classic” structural lock-and-key mechanism, originally based on surveys conducted in Diptera [41] (Figure 1), the co-evolved firmly sclerotised structures should be responsible for correct species-specific copulation, as shown by numerous microscopic surveys, e.g., in different species of Geometrids and Noctuid moths [19,40], and as recently shown experimentally in Lygaeidae bugs [42].

(ii) According to the sensory lock-and-key mechanism [43,44], special structural traits in genital morphology evoke behavioural/physiological responses that promote the successful copulation and fertilisation.

However, as the external genital appendages and the internal genitalia have different forms of sclerotisation and are associated with different functions and phases of copulation, approaches (i) and (ii) do not exclude each other but they can be combined or allied in a multi-step process.

**Figure 2:**
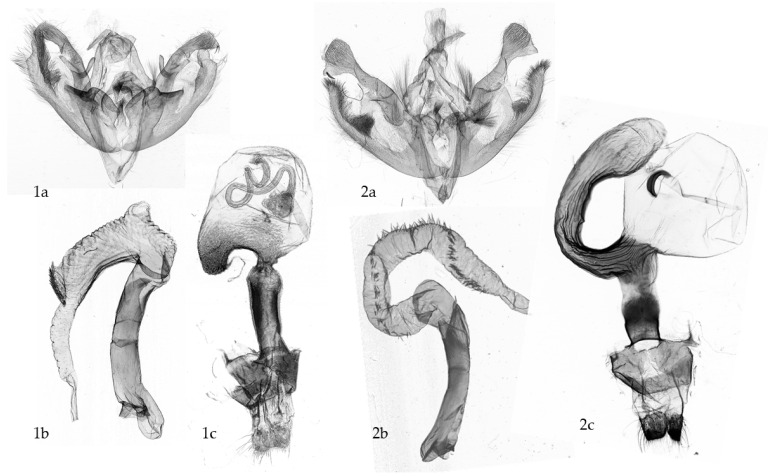
Examples of secondary asymmetry of the genital capsula, with simple vs. sophisticated forms of the “lock-and-key” structure of the inner genitalia of *Polia* spp. (Hadenini). (**1a**–**c**) *Polia (Polia) vesperugo* Eversmann, 1856 ((**a**), male genital capsule; (**b**), male endophallus (vesica) (**c**), female genitalia); (**2a**–**c**) *Polia (Metallopolia) metagnorima* Varga, G. Ronkay, L. Ronkay, 2018 ((**a**), male genital capsula; (**b**), male endophallus (vesica); (**c**), female genitalia).

Functionally, two different components can be distinguished. The sensory lock-and-key system comprises special traits in genital morphology, as the androconial tufts (coremata) on different parts of the abdomen and/or external genitalia, and specialised types of bristles on different parts of the external genitalia (syn.: genital capsule, Figure 1), which function mostly in the courtship phase of sexual contact [40,42]. The other component is the co-adapted and co-evolved endophallus–bursa copulatrix complex, which comes into action following firm contact between external genitalia [19,36] and is responsible for the correct transfer and allocation of the spermatophore (Figure 2). Therefore, the often discussed “lock-and-key” mechanisms operate during both the pre-mating and mating steps of copulation.

The external genitalia consist of modified sternits, tergits, and gonopodia both in Diptera and Lepidoptera (Figure 1). It is accepted that they function in pre-insemination sexual selection [45], and it has been suggested that lock-and-key mechanisms of the sclerotised external genitalia effectively contribute to reproductive isolation (RI). However, questions have been raised regarding the fact that despite the high diversity of genital structures in animal taxa, the external genitalia do not cause structural lock-and-key RI in a strict sense [46]. Therefore, it is necessary to consider the structure and function of both the external and internal genitalia.

As a result of the revision of numerous polyandrous species in the subtribe Poliina (Noctuidae), it was shown that the evolution of secondary asymmetry in male external genitalia appears to be connected with an increase in species diversity [47,48]. Therefore, we hypothesised that the asymmetric allocation of sensory and stimulating functions on the external male genitalia can enhance sexual selection and thus speciation. The other aspect of sexual selection is the interaction between the external and internal genitalia, i.e., the possible evolutionary trade-off associated with external vs. internal genitalia. Therefore, we hypothesised that a simplified genital capsule is associated with a sophisticated internal lock-and-key structure and vice versa.

This hypothesis was tested in some genera of Hadenini (Noctuidae, Noctuinae). We have demonstrated that the two most diverse genera, *Polia* and *Ctenoceratoda* (Poliina subtribe), display two divergent trends of change originating from the bilaterally symmetrical structures of the genital capsula.

(α) In *Polia*, the enhanced complexity of the external genitalia was found to be connected with secondary asymmetrisation of the functionally active parts (clasping, stimulation, and sensual function) and combined with less complex structures of the inner genitalia, i.e., an opposite trend was shown in the complexity of the external vs. internal genitalia (Figure 2).

(β) Oppositely, in *Ctenoceratoda*, the simplification of genital capsula was associated with a trend of sophistication of the internal genitalia in both sexes, which resulted in the co-evolved “lock-and-key” system of the vesica seminalis and bursa copulatrix [49].

**Figure 3:**
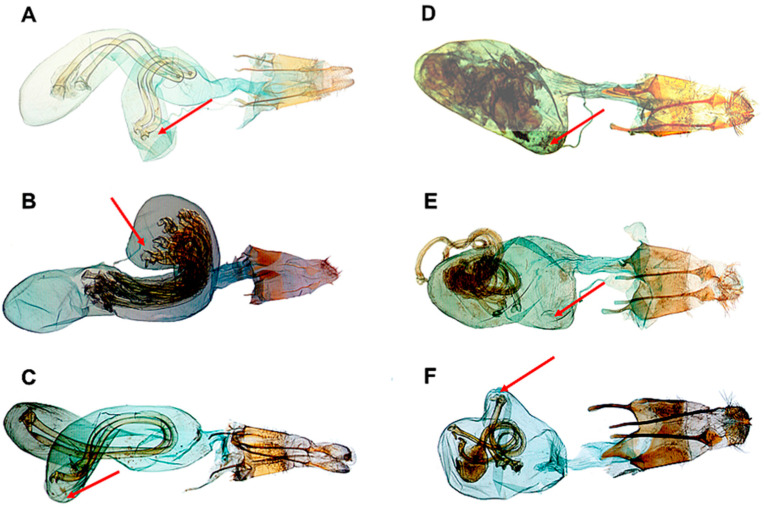
Examples of polyandrous species from the “pest clade” of Noctuidae, Noctuini. Slides of female genitalia with different numbers of spermatophores in bursa copulatrix [in brackets: provenance of specimen, number of permanent slides in Euparal, **number of spermatophores (bold)].** (**A**): *Dichagyris devota* (Christoph, 1884) [Turkmenistan, Kopet dagh Mts. VZ 9452 (**2**)]; (**B**): *Dichagyris melanura* (Kollar, 1846) [Greece, Olymp Mts. VZ 9429 (**4**)]; (**C**): *Dichagyris forficula* (Eversmann, 1851) [Turkey, Ziyaret gecidi, VZ 10,223 (**2**)]; (**D**): *Euxoa decora olympica* Tuleshkov, 1951 [Greece, Olymp Mts. VZ 11,438 (**7**)]; (**E**): *Euxoa transcaspica* Kozhanchikov, [Turkmenistan, Kopet dagh Mts. VZ 9067 (**3**)]; (**F**): *Euxoa tristis* (Staudinger, 1898) [Mongolia, Mongol Altai Mts. VZ 10,226 (**2**)] (Slides: Z. Varga).

### 3.2. Preliminary Results: Correlates and Consequences of Polyandry

Surveys investigating the reproduction physiology of moths have shown that mating is a time-consuming, multi-phase act, as demonstrated by laboratory experiments on the polyandrous moths *Spodoptera litura* (Noctuidae [18]) and *Ephestia kuehniella* (Pyralidae [50]). As visualised in microscopic studies [39,47], the co-evolved matching of the inner genitalia results in proper lodging of the spermatophores into the bursa, i.e., the aperture of the spermatophore was shown to be oriented to the appendix bursae, near to the ductus seminalis [35,51]. We have found the same results in numerous species of the multi-diverse and polyandrous genera *Euxoa* and *Dichagyris* (Noctuidae, Noctuinae; Figure 3A–F).

Another relevant aspect of the reproductive biology of moths is the number of matings within the life-span, which can be experimentally determined by visual observation or using video-cameras. A different, simpler method is to dissect females and count the number of spermatophores (Figure 3A–F). In this survey, we took the latter approach and dissected a large number of females collected using light and volatile traps. We confirmed that a large number of species of the “pest clade” of Noctuidae (subf. Noctuinae) are usually polyandrous (Figure 3 and Supplementary Information).

**Figure 4:**
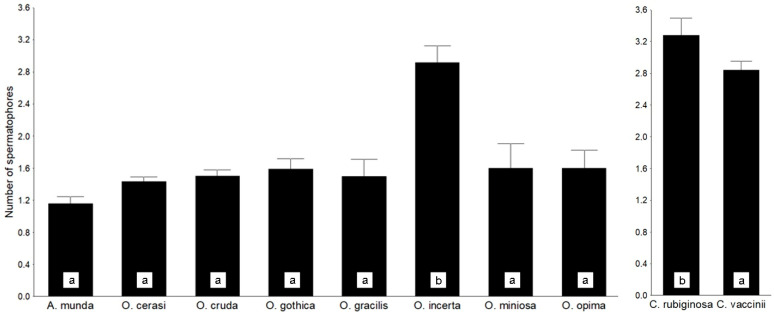
Level of polyandry in *Orthosia* (s.l.) and *Conistra* species (data: Supplementary Materials). Means followed by different lowercase letters differ significantly (*p* < 0.05).

However, the level of polyandry varies considerably at the individual, population, and species levels. In the phenologically nearly synchronic and moderately diverse genus *Orthosia* (Noctuidae, Hadenini), we found that the externally highly variable *O. incerta* seem to be mostly polyandrous. The other species in this genus proved to be only moderately polyandrous, without significant differences, while the more distantly related *Anorthoa munda* seems to be the least polyandrous (Figure 4, Supplementary Materials).

We also found that two common species of *Conistra* display a much higher level of polyandry (Figure 4, Supplementary Materials), despite the similarity of their reproduction periods to that of *Orthosia* spp., incl. copulation and oviposition, which are adapted to the bud-bursting time of broadleaved trees such as *Quercus* spp. and *Carpinus*. However, *Conistra* species have an entirely different life cycle, including the hibernation of adult moths [52].

## 4. Discussion and Remaining Questions

### 4.1. Connection of Polyandry to Life Cycle: Hypotheses and Preliminary Results

Based on our preliminary data, we attempted to formulate some hypotheses regarding the correlates and consequences of polyandry in connection to life cycle. One of the first questions relates to the connection of the polyandry with fertility and/or fecundity. According to a review by Torres-Villa et al. [16], in most polyandrous Noctuidae species (9 from 12), remating significantly increased the fertility of females under experimental conditions. Thus, they hypothesised that polyandry evolved with and/or is maintained by the improved fertility of females. However, they also mentioned that the outputs of their experiments were biased, since preferably polyandrous species were selected for the analyses. It was also shown that some closely related species seem to react differently, e.g., higher fertility was found in *Helicoverpa armigera* vs. *Heliothis virescens* and in *Spodoptera exigua* vs. *S. litoralis* [18,23,24,25].

In an earlier publication, the question of fertility was connected with the concept of the r vs. K strategy. However, in this case, fecundity was measured as the number of eggs produced by females. In Hadeninae species, Spitzer et al. (1984) [53] found that in *Orthosia,* as well as some other non-closely-related species (e.g., *Mythimna* spp., *Cerapteryx graminis*, *Tholera decimalis*), the ovaria of dissected females contained only medium to high numbers of eggs (137–378). Similarly, lower egg numbers (92–215) were found in several habitat specialists, e.g., in some wetland species such as *Apamea ophiogramma*, *Photedes pygmina*, *Hydraecia micacea*, and *Celaena leucostigma*. All these species are known to be strictly univoltine. In contrast, most migrating and/or pest species proved to be r-strategists, based not only on the significantly higher numbers of eggs observed (700–4000) but also on their plasticity in voltinism (e.g., *Agrotis segetum*, *Noctua pronuba*, *Lacanobia oleracea*, *Autographa gamma*). However, these studies did not clarify whether the higher fecundity of these bi- or multivoltine migrating species relates to the higher level of polyandry or if these traits vary independently. A similarly understudied topic is the connection between polyandry and longevity or, more precisely, whether polyandry occurs over the whole life-span of imagoes or is restricted to a period (e.g., after hibernation, as noted in *Conistra, Eupsilia* spp., or following aestivation, as in some Noctuinae species, e.g., in the species of the *Chersotis capnistis* group [54]).

The other crucial question is the connection of polyandry and sexual selection. Competition among males to access multiple mates was hypothesised to be the main driver of sexual selection (Darwin–Bateman paradigm, ref. [7]). Sexual selection can occur according to female choice in the pre-mating phase or through cryptic choice, when the sperm is either used for fertilisation or digested as an energy source (“nuptial gift”) [55]. Since polyandry generates competition among females, females should benefit from polyandry by enhanced fertility and thus improved fitness [3,16]. Supposedly, if female remating behaviour results in higher individual fitness due to increasing fertility, than the genes being responsible for disposition to remating will be selected for and dispersed in the population by gene flow.

Since polyandry implies competition, we should ask, how is selection connected with competition? We have seen that both competition and selection act at different levels, since copulation and fertilisation are multi-step processes. Therefore, we conclude that the lock-and-key structures also function at more levels, i.e., the mechanic and sensory external structures are responsible for “pre-insemination” sexual selection [40]. The next step of sexual selection is connected to the co-evolution of the internal genital structures, since these are responsible for the correct transfer and allocation of the spermatophores and thus for the possibility of sperm competition among the ejaculates of different males. We hypothesised that serial polyandry can promote competition, but visual observation alone proved insufficient for the demonstration of the pre-copulatory selection. Therefore, the hypothesis was experimentally tested [56]. Unfortunately, the time-sequenced dissections and counting of spermatophores in the bursa copulatrix, following the methods of Callahan [39], are insufficient to show the last step of sexual selection, i.e., the result of the female choice.

The latitudinal cline in the frequency of remating was studied in laboratory populations of *Drosophila pseudoobscura.* It was found that females of all genotypes remated more frequently at cooler temperatures [35]. This result agrees with field observations, which have shown higher average frequencies of polyandry at higher latitudes. Thus, it is likely that genetic factors are responsible for the latitudinal cline in polyandry. In such cases, the level of polyandry should be different in uni- vs. bi-(or poly) voltine populations of the same species, as was shown in the polyandric White *Pieris napi* [57]. However, these case studies did not lead to any general conclusions regarding the genetic background of polyandry.

### 4.2. Questions and Suggestions for Further Discussion

An open question remains regarding is the geographical distribution of polyandry. It was hypothesised that polyandry is more common in northern latitudes [1,35]. Supposedly, females have a better chance for longevity in the North due to lower temperatures, and this circumstance provides more opportunities for females to remate. An other possibility may be that moths resume activity at cooler temperatures, which has been observed up in many Noctuidae after aestivation, e.g., in *Euxoa sibirica* [56]. However, such data on correlations alone cannot support any hypotheses about causality.

Many other important questions remain understudied. We do not have sufficient data, e.g., on the connection between the sex ratio and level of polyandry—are they positively correlated? Another fascinating subject is the possible interaction of the male-biased sex ratio with the level of eupyrene vs. apyrene sperm (sperm with vs. without nuclear genes) since apyrene sperm could serve as a “nuptial gift”, i.e., an energy source for greater fertility. In this context, we also need to clarify the role of endosymbiotic *Wolbachia*. This means that the question should be re-formulated: does *Wolbachia* function as a factor of sexual selection through the manipulation of the eupyrene/apyrene sperm ratio and/or through the manipulation of the sex ratio? All these questions may be of vital importance to not only understanding the population dynamics of pest and/or migrating species but also the conservation of threatened butterfly and moth species.

To answer these questions, we need much more reliable data on polyandry and sex ratios in moth populations, since there is a supposed interaction between sex ratio and polyandry. If the sex ratio is male-biased, it should imply a higher level of polyandry. Therefore, the results obtained from heavily sex-based samplings, e.g., those that use pheromone or light traps, may be misleading. To avoid this imbalance, we suggest that in the interest of correct and comparable sampling, the recently developed bisexual methods of trapping, e.g., volatile traps, should be used, based on experiments in which phenylacetaldehyde and/or natural floral volatile materials were successfully used to attract high numbers of both sexes of several Noctuid pest species [58,59,60].

## 5. Conclusions

Our surveys on several Noctuid species have shown that polyandry is a regular phenomenon in these insects. It is relatively common in species of some economically important genera but also displays close connections with some life history traits, such as hibernation in the imago state.

According to data published in multiple studies (e.g., refs. [16,20,23,24,25]), polyandry has been observed in phylogenetically different genera of Noctuidae, in both migrating species, such as Heliothidinae, Plusiinae, and in pest species of Xyleninae (*Spodoptera*). It has also been observed in some more sedentary habitat specialists from grasslands (e.g., *Euxoa, Dichagyris* spp.) and woody habitats (e.g., *Orthosia, Conistra*, and related genera). Since the species of these genera have different life cycles, future studies should determine whether ecology and/or phylogeny influence the level of polyandry.

Our results confirm that competition and selection act at different levels, since copulation and fertilisation are multi-step processes; thus, the lock-and-key structures also function at multiple levels. Our results seem to support the hypothesis that co-evolved internal genital structures, which are responsible for the correct allocation of the spermatophores, are responsible for sexual selection due to the possibility of sperm competition among the ejaculates of different males. However, the most important question remains unanswered: whether polyandry and fecundity are interconnected; in other words, how can polyandry influence or even improve fitness, and how are these traits reflected in population frequency cycles, particularly in pest species?

## Data Availability

The datasets generated during and/or analysed during the current study are available in the ZENODO repository: Varga, Z., Nagy, A., Kovács, L.C., Szanyi, Sz. (2024). Polyandry in Lepidoptera, ecological and evolutionary implications—preliminary review—RAW data [Data set]. Zenodo. https://doi.org/10.5281/zenodo.12630092.

## Supplementary Materials

The following supporting information can be downloaded at: https://www.mdpi.com/article/10.3390/insects16101063/s1, Figure S1: Supplementary material_examples; Table S1: Supplementary material_TABLE.
