# Supplementary material for: Polyandry in Noctuid Moths: Taxonomic, Bionomic, and Evolutionary Implications
Source: Insects. 2025 Oct 17;16(10):1063. doi: 10.3390/insects16101063 (PMC12564964; doi:10.3390/insects16101063)
Supplement: Supplementary file 1 [file insects-16-01063-s001.zip › Supplementary material_examples.pdf]

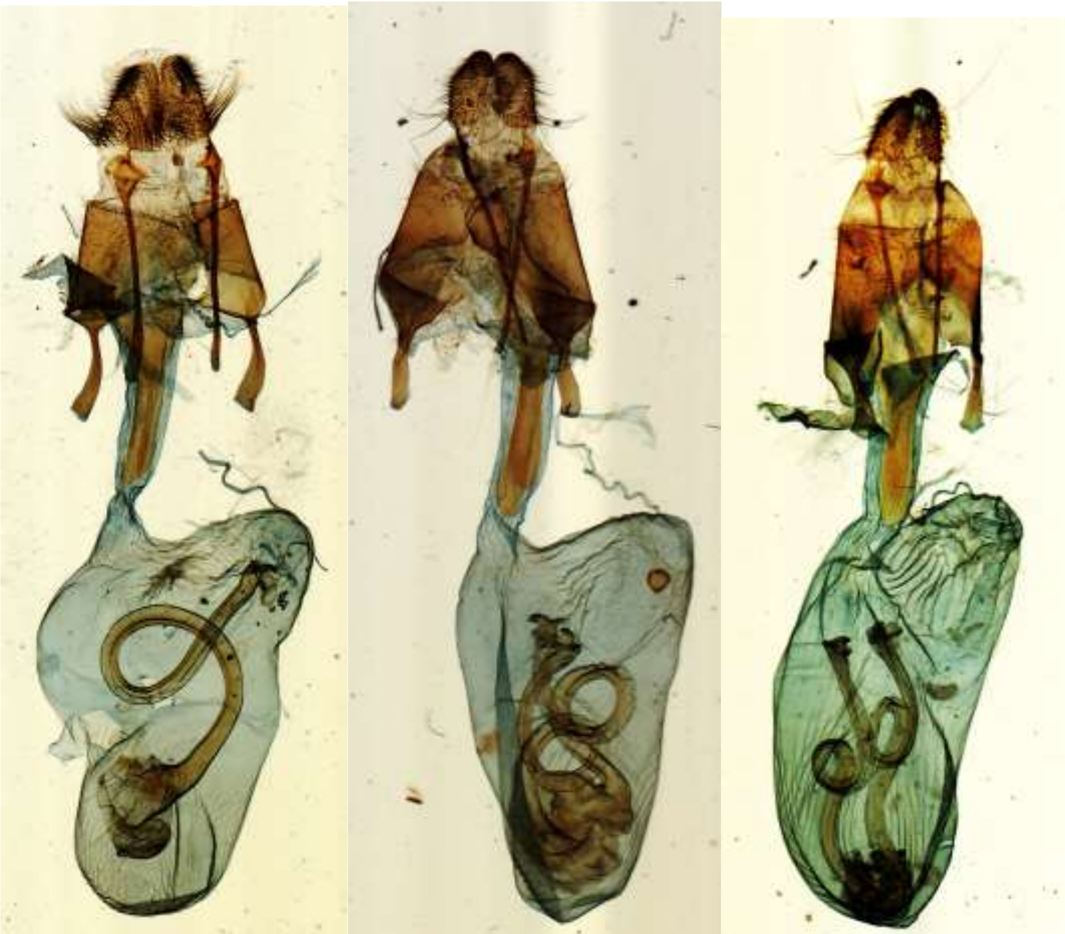

1. Euxoa dichagyroides Varga Pakistan, Hindukush Mts. VZ11290 (**1**), 2. Euxoa fallax Ev. Kazakhstan, Kapchugay VZ10957 (**2**), 3. Euxoa diaphora Boursin Russia, Sarepta VZ11328 (**2**)

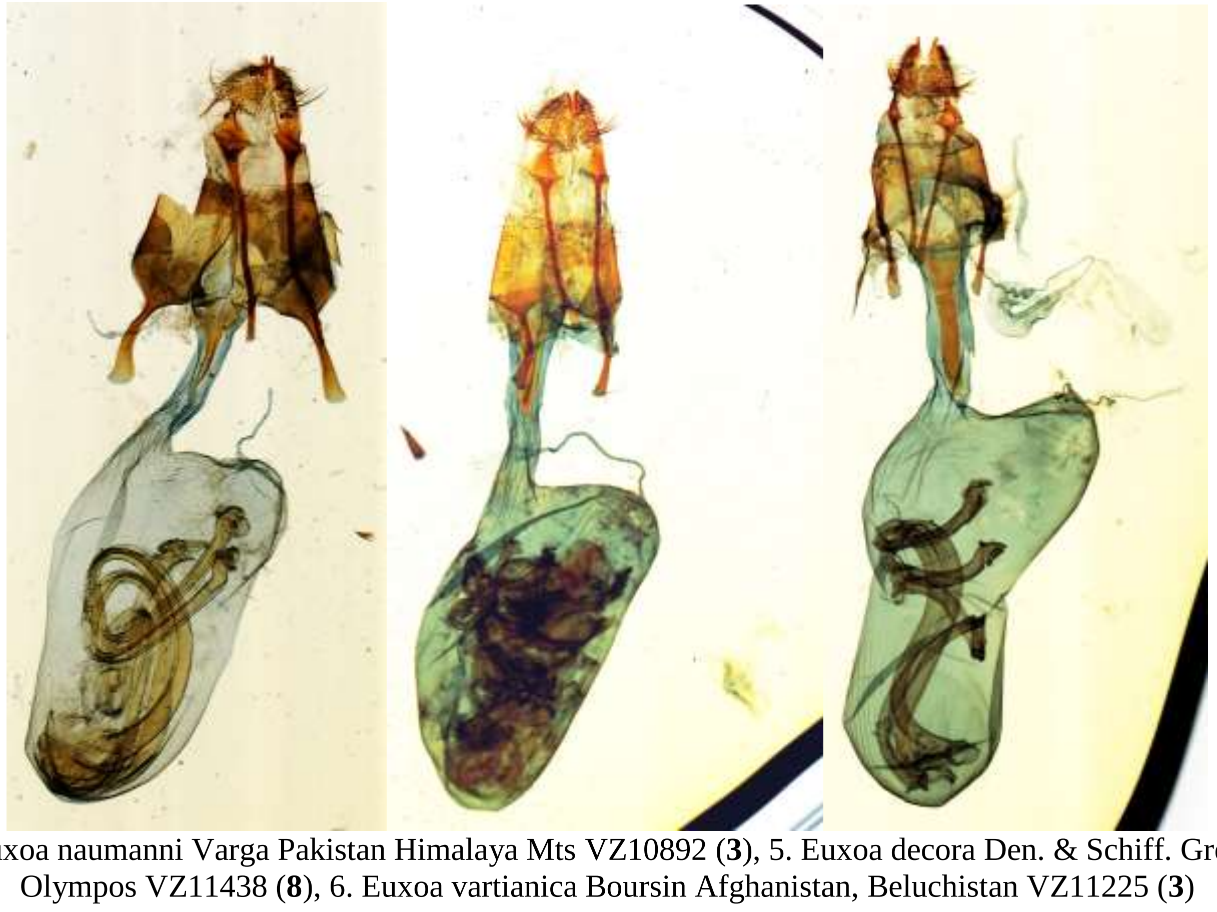

4. Euxoa naumanni Varga Pakistan Himalaya Mts VZ10892 (**3**), 5. Euxoa decora Den. & Schiff. Greece, Olympos VZ11438 (**8**), 6. Euxoa vartianica Boursin Afghanistan, Beluchistan VZ11225 (**3**)

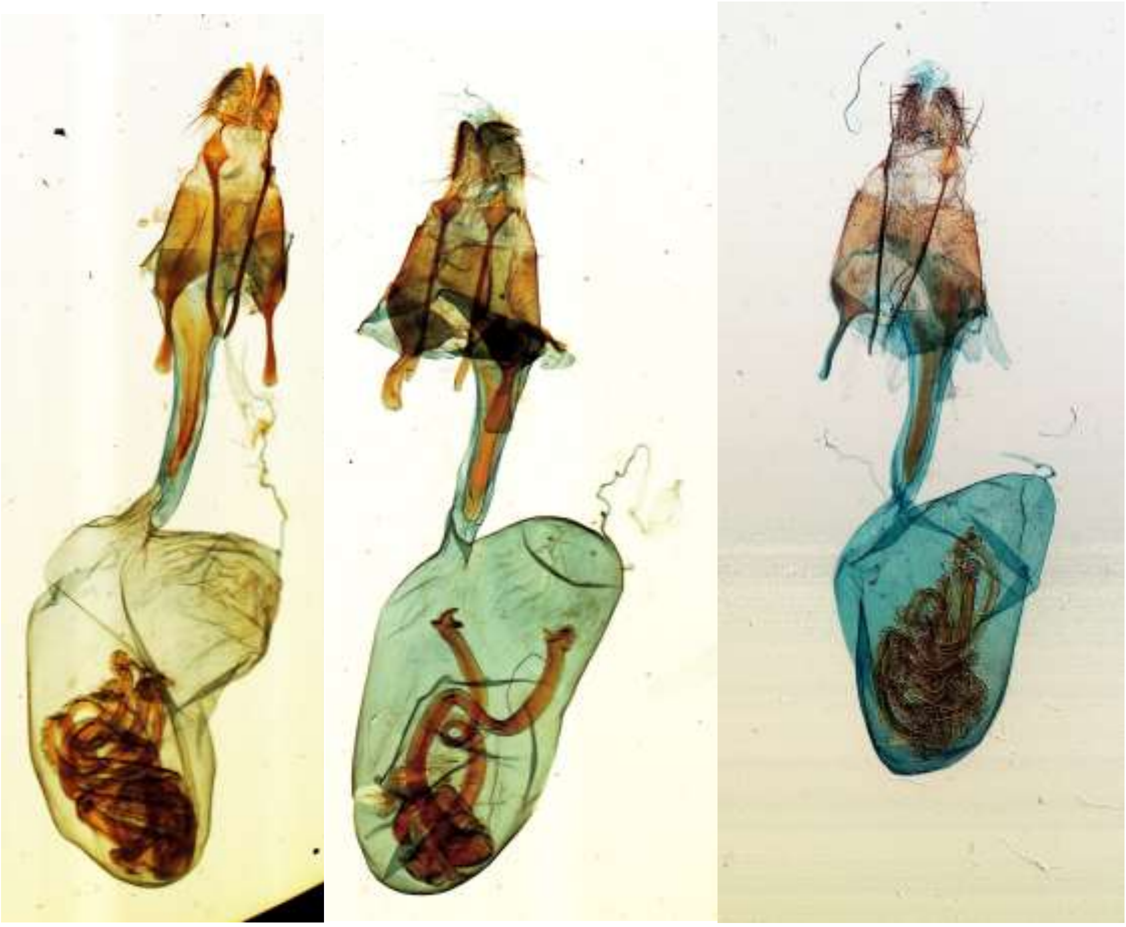

7. Euxoa birivia Den. & Schiff. Turkey prov. Agri VZ6168 (**4**), 8. Euxoa seliginis Gn. Kazakhstan, Kapchugay VZ11832 (**2**) 9. Euxoa cespitis Sw. Afghanistan, Dasht-i-Nawar VZ9008 (**4**)

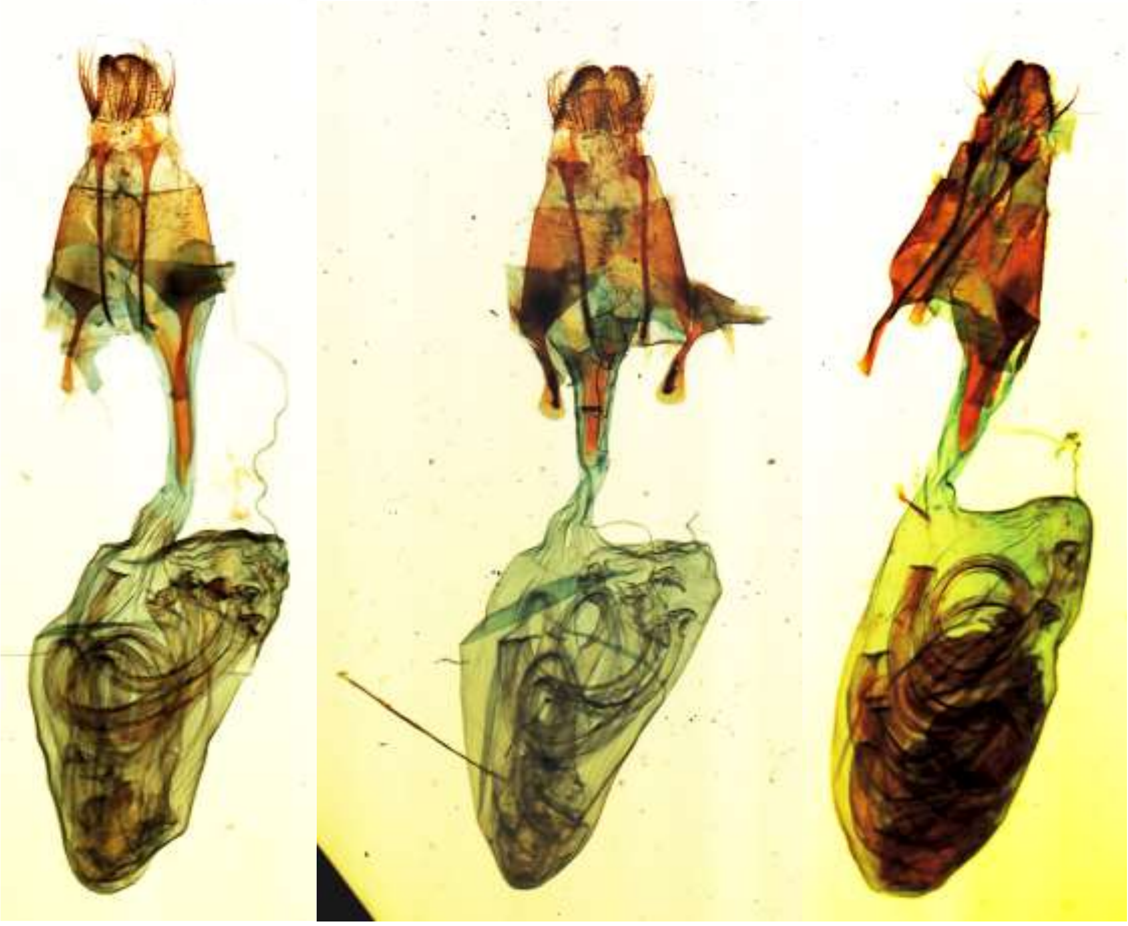

10. Euxoa aquilina Den. & Schiff. Uzbekistan, Mt. Chimgan VZ11080 (**7**), 11. Euxoa aquilina Den. & Schiff. Turkey, Akshehir VZ11078 (**5**) 12. Euxoa aquilina Den. & Schiff. Turkey, prov. Van VZ11079 (?**9**)

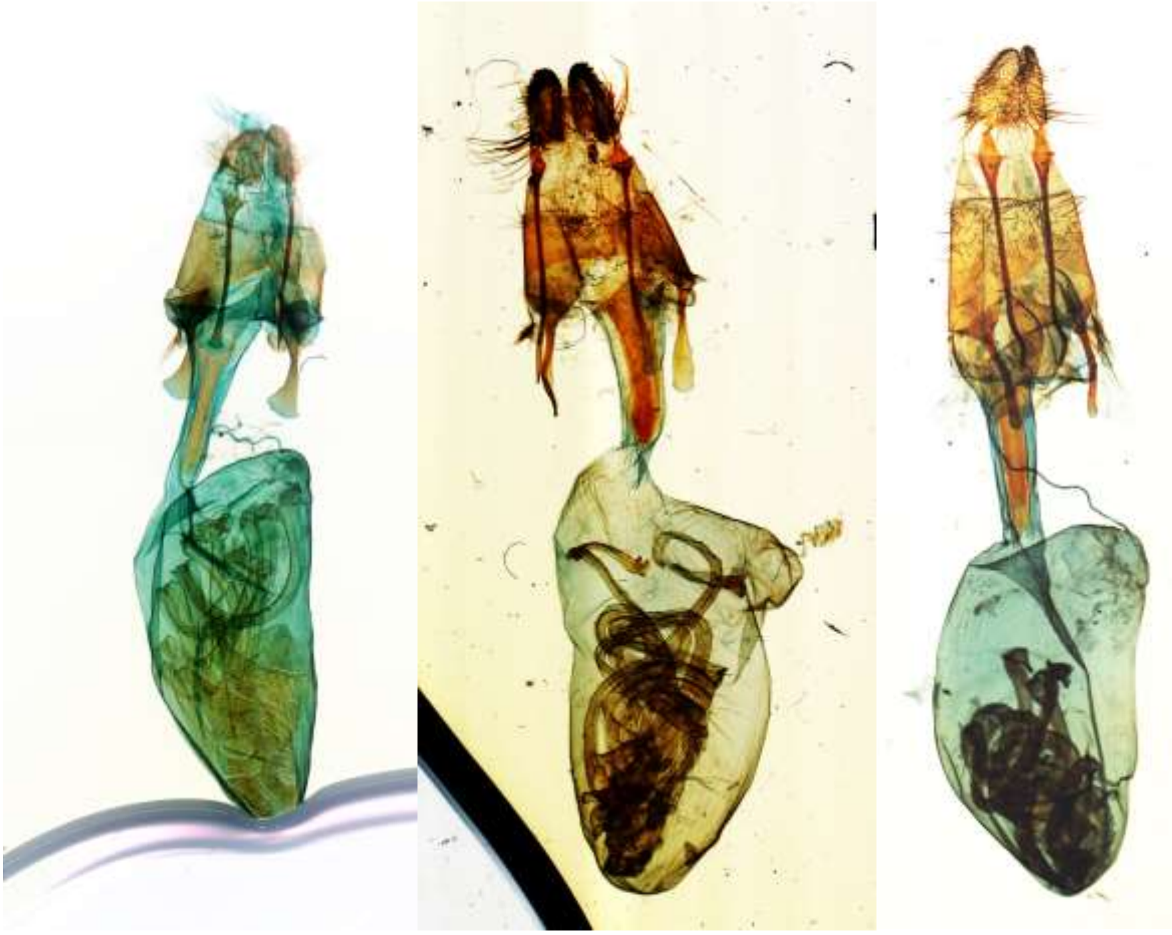

13. Euxoa eruta Esp. HU Debrecen VZ7287 (7), 14. Euxoa homicida Stgr. Turkey, prov. Konya VZ10873 (**4**), 15. Euxoa robiginosa Stgr. Turkey prov. Konya VZ10959 (**3**)

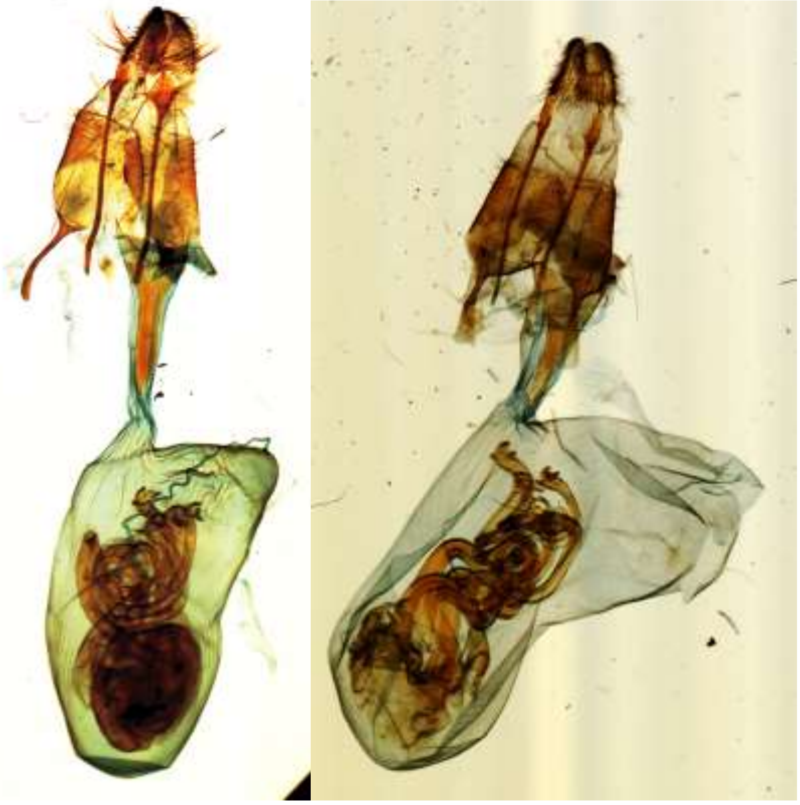

16. Euxoa aquilina Den. & Schiff. Turkey prov. Agri VZ10961 (5),
17. Euxoa (Pleonectopoda) derrae Hacker Greece, Phalakron Oros VZ10893 (**4**)

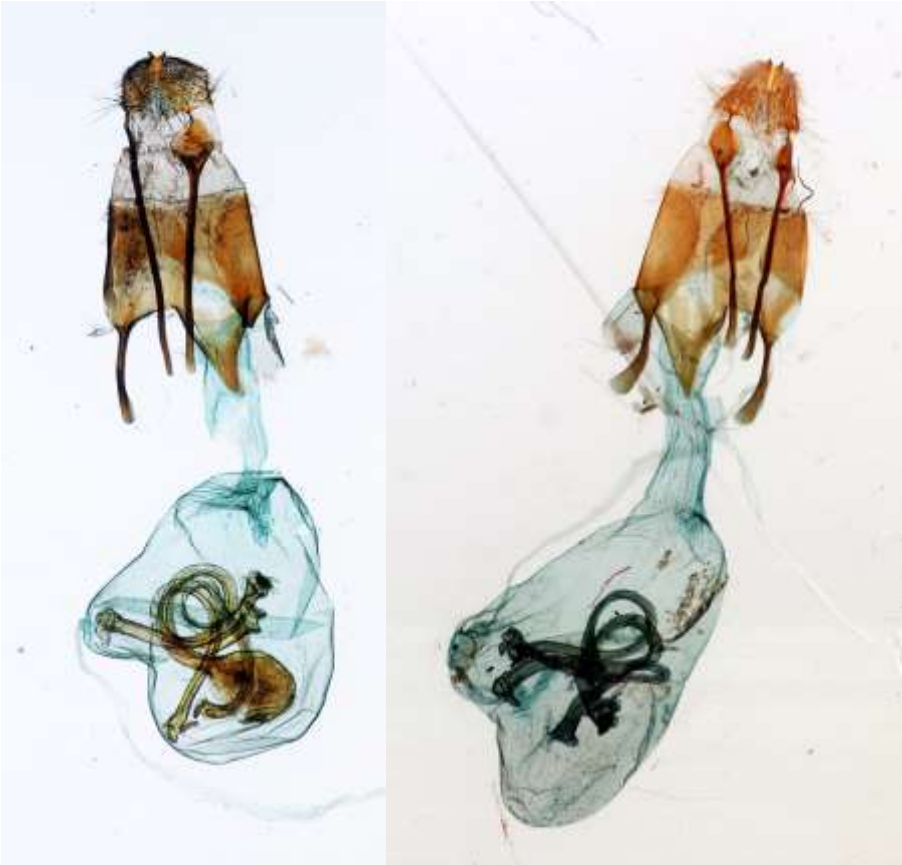

18. Euxoa (Orosagrotis) tristis Stgr. Mongolia Chovd aimak VZ10226 (**3**)
19. Euxoa (Orosagrotis) tristis Stgr. Mongolia, Bulgan aimak VZ10322 (**4**)

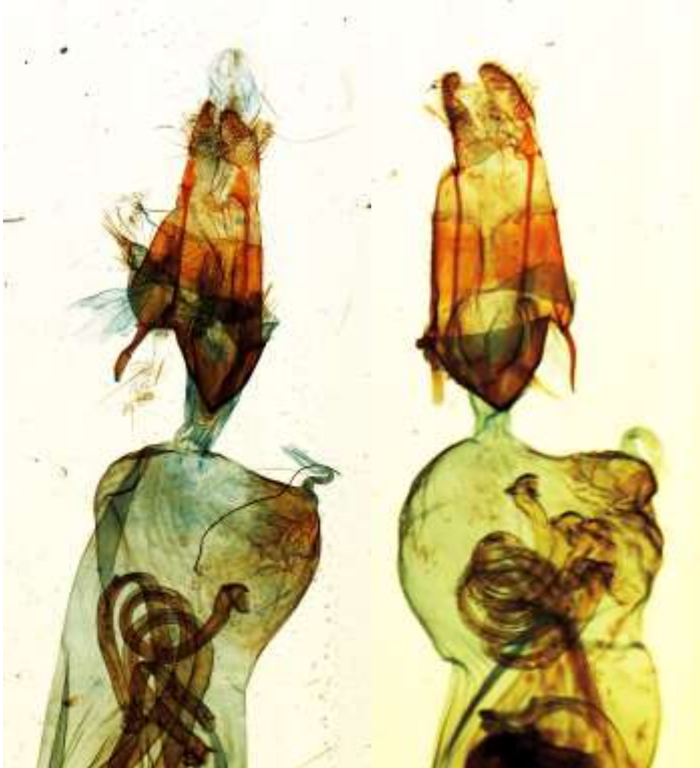

20. Euxoa (Chorizagrotis) adumbrata Ev. Nepal, Annapurna Himal, VZ10508 (**3**)
21. Euxoa (Chorizagrotis) inexpectata Alph. India, Himachal Pr. VZ11325 (**4**)
