# Supplementary material for: Polyandry in Noctuid Moths: Taxonomic, Bionomic, and Evolutionary Implications
Source: Insects. 2025 Oct 17;16(10):1063. doi: 10.3390/insects16101063 (PMC12564964; doi:10.3390/insects16101063)
Supplement: Supplementary file 1 [file insects-16-01063-s001.zip › Supplementary material_TABLE.pdf]

Supplementary material

Species	N	Min. number of spermatophore/ind	Max. number of spermatophore/ind	Mean	Std.Dev.	Std.Err	-95,00%	+95,00%
Anorthoa munda	19	1	2	1,157895	0,374634	0,085947	0,977327	1,338463
Orthosia cerasi	76	1	3	1,434211	0,524990	0,060220	1,314245	1,554176
Orthosia cruda	115	1	4	1,504348	0,820504	0,076512	1,352777	1,655918
Orthosia gothica	34	1	4	1,588235	0,743360	0,127485	1,328865	1,847606
Orthosia gracilis	20	1	5	1,500000	0,945905	0,211511	1,057303	1,942697
Orthosia incerta	60	1	7	2,916667	1,618659	0,208968	2,498523	3,334811
Orthosia miniosa	10	1	4	1,600000	0,966092	0,305505	0,908900	2,291100
Orthosia optima	10	1	3	1,600000	0,699206	0,221108	1,099818	2,100182
Conistra rubiginosa	47	1	6	3,276596	1,513736	0,220801	2,832146	3,721045
Conistra vaccinii	158	1	9	2,841772	1,398462	0,111256	2,622021	3,061523
